# Considering ethical issues in evaluation studies on remote patient monitoring for cancer and cardiovascular diseases: a scoping review

**DOI:** 10.3389/fdgth.2026.1905222

**Published:** 2026-10-30

**Authors:** Linda Wienands, Sabine Valenta, Lynn Leppla, Sabina De Geest, László Kovács, Alexandra Teynor, Julia Krumme

**Affiliations:** 1 Department of Nursing, Medical Center – University of Freiburg, Faculty of Medicine, University of Freiburg, Freiburg im Breisgau, Germany; 2 Nursing Science, Department Public Health, Faculty of Medicine, University of Basel, Basel, Switzerland; 3 Nursing Science, Institute for Health Sciences, Faculty of Medicine, University of Tübingen, Tübingen, Germany; 4 Department of Medicine I, Medical Center – University of Freiburg, Faculty of Medicine, University of Freiburg, Freiburg im Breisgau, Germany; 5 Academic Center for Nursing and Midwifery, Department of Public Health and Primary Care, KU Leuven, Leuven, Belgium; 6 Faculty of Liberal Arts and Sciences, Technical University of Applied Sciences Augsburg, Augsburg, Germany; 7 Faculty of Computer Science, Technical University of Applied Sciences Augsburg, Augsburg, Germany

**Keywords:** cancer, cardiovascular diseases, digital health, eHealth, ethical issues, ethics, evaluation, remote patient monitoring

## Abstract

**Introduction:**

Considering ethical issues is increasingly recognized as a core component of robust digital health evaluations. However, no agreed-upon standard guides evaluators on how such issues should be systematically identified, examined, and reported. Moreover, the extent to which ethical issues are addressed in digital health evaluation studies remains unclear, as existing reviews have not examined this question in sufficient depth. This scoping review aims to address this gap by exploring how ethical issues are addressed and reported in evaluation research on remote patient monitoring (RPM) technologies in cancer and cardiovascular disease care.

**Methods:**

This scoping review was conducted in accordance with the Joanna Briggs Institute methodology and reported following the PRISMA-ScR guidelines. MEDLINE, Embase, CINAHL, PsycINFO, SocINDEX, and the Philosopher's Index were searched, complemented by a Google Scholar search for additional peer-reviewed studies. Eligible sources included original evaluation studies of non-fully automated RPM requiring active patient or caregiver engagement and bidirectional interaction with healthcare providers in cancer and cardiovascular disease care. A protocol-based extraction template was iteratively refined, and a mixed deductive–inductive approach was applied. Data were analyzed using basic qualitative content analysis.

**Results:**

Ethically relevant issues were grouped into four analytical categories: *Integrity in Research and Development*, *Ambivalent Impacts of RPM*, *Justice and Equity*, and *Data Privacy and Security*. Across these categories, ethically relevant issues were frequently reported, but their treatment often remained descriptive, while normative or critical reflection on their ethical implications was much less visible. Explicit references to ethics were largely confined to statements of ethics approval, providing little insight into the underlying ethical reflection. Furthermore, an emphasis on positive findings was identified, while reflections on the ethical implications for non-beneficiaries remained limited. Similarly, reporting of data privacy and security measures related to the evaluated digital health tools was often insufficient.

**Discussion:**

Ethically relevant issues were evident, but explicit and systematic reflection on their ethical implications was less visible in the reporting. More reflective ethical engagement, supported by interdisciplinary collaboration and shared empirical-ethical guidance, may foster more ethically informed digital health research and practice.

## 1 Introduction

Evaluation research plays a key role in shaping digital transformation in healthcare, which is being pursued worldwide (1). Its findings guide decisions about whether and how digital health tools are developed and implemented, and which receive funding, reimbursement, or are considered for broader adoption (2). This places considerable responsibility on evaluators, whose work should extend beyond empirical and technical assessments. They need to engage with ethical issues to ensure that evaluations consider not only effectiveness, but also questions of safety, fairness, inclusiveness, and broader societal implications (3–5).

A prominent example of this digital transformation is the use of remote patient monitoring (RPM) technologies in healthcare. RPM entails the collection of patients' health data outside traditional clinical settings and their transmission to healthcare providers for assessment (6). By enabling patients and clinicians to continuously track health indicators over time, RPM may support a more holistic understanding of health trajectories and facilitate the early detection of deterioration (6, 7). This may be particularly relevant in severe illnesses such as cancer and cardiovascular disease, where timely intervention can help prevent complications (8, 9).

However, digital health technologies such as RPM not only offer potential benefits but also raise a range of ethical issues. These include concerns about data privacy and equitable access to technology, as well as the risks of unintended harm and infringements on patient and clinician autonomy (10–15). Additional ethical issues may arise when RPM systems incorporate automated functions and algorithm-based decision support. These developments introduce uncertainties regarding the accuracy, reliability, and transparency of system outputs. They also raise questions about responsibility and accountability when clinical decision-making is influenced by such systems (16, 17). Given the wide range of ethical issues associated with RPM, there is growing recognition across the academic literature, institutional frameworks, and government policy that these issues must be addressed in digital health (15, 18–22).

In response to these challenges, applied ethics offers procedural and normative guidance for the systematic consideration of ethical issues in professional contexts (23). In digital health, this includes overarching ethical approaches (e.g., moral theories, principlism, and discourse ethics) and more recent frameworks developed specifically for digital health contexts and related processes, including technology development and research (17, 24–34). Importantly, within these contemporary digital health-specific approaches, ethical reflection is not understood as an activity reserved for ethicists alone but as an integral part of everyday professional practice (27, 33, 35). In the context of evaluation research, empirical and ethical perspectives can complement one another (see Figure 1): ethical reflection on what OUGHT to be can inform empirical evaluation practice, while empirical findings about what IS can, in turn, inform ethical reflection. Together, these perspectives can support conclusions and recommendations for ACTION that are empirically and ethically justified (17, 33, 35, 36).

**Figure 1:**
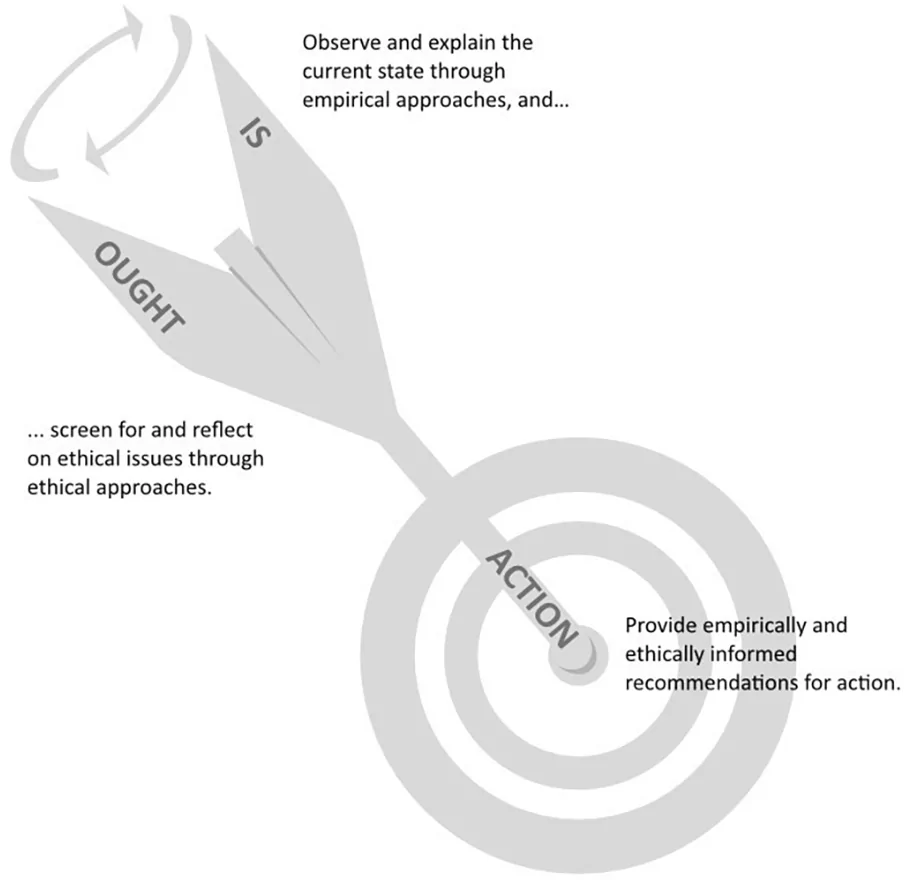
Digital health evaluation research incorporating empirical and ethical perspectives (36).

Although there is growing consensus on the need to integrate empirical and ethical perspectives, substantial methodological challenges remain. Established digital health evaluation approaches, including guidance from the World Health Organization (WHO) (37), Medical Research Council (38, 39), and the RE-AIM (Reach, Effectiveness, Adoption, Implementation, Maintenance) framework (40), provide little explicit guidance on how ethical reflection should be integrated into empirical evaluation. Conversely, ethical frameworks rarely specify how ethical reflection can be incorporated into established empirical evaluation methods, and there is no consensus on a single appropriate ethical approach (41). Consequently, it remains unclear when and how digital health-specific ethical issues should be addressed, how ethical reflection can be integrated across different study designs, and how such reflection should ultimately be reported transparently. Clearer shared guidance is therefore needed to better connect ethical reflection with empirical evaluation.

Previous reviews suggest limited attention to ethics in digital health research (13, 42–49), but their findings are difficult to compare because they differ in what they consider ethically relevant and how they identify ethical content. Some include research process-related aspects (42), while others do not (13, 48). Moreover, many rely on keyword-based approaches that identify explicit references to “ethics” (13, 42–49), potentially overlooking ethically relevant content that is not explicitly framed as ethical. A feasibility study by Appleyard et al. (50) illustrates this limitation. Although the authors did not explicitly frame these issues in ethical terms, they addressed digital exclusion among older cancer patients and recommended a more inclusive RPM design, thereby addressing issues related to justice and equity. Classifying such work as not addressing ethics could therefore reinforce a narrow understanding of ethics as an external add-on rather than an integral part of evaluation practice. Consequently, existing reviews provide limited insight into which ethically relevant issues are addressed in evaluation research, how they are addressed, and whether studies move beyond descriptive reporting to reflect on their ethical implications. To address these shortcomings, a clearer understanding of which ethically relevant issues are addressed in original digital health evaluation research is needed, whether or not they are explicitly framed as ethical. This scoping review systematically identifies and maps ethically relevant issues in RPM evaluation studies and examines how the studies address them. Focusing on RPM in cancer and cardiovascular care, this review highlights both gaps and opportunities to strengthen how ethical issues are addressed in future evaluation research.

This review was guided by three research questions:

1. To what extent do original digital health evaluation studies explicitly or implicitly address ethically relevant issues?
2. Which ethically relevant issues are addressed, and how are they handled methodologically?
3. What shortcomings and promising starting points can be identified to enhance ethical engagement in future evaluation research?

## 2 Materials and methods

This scoping review was conducted in accordance with the Joanna Briggs Institute (JBI) methodology for scoping reviews (51) and reported following the Preferred Reporting Items for Systematic Reviews and Meta-Analyses extension for Scoping Reviews (PRISMA-ScR) guidelines (52). The review was preregistered on the Open Science Framework (OSF) under https://osf.io/7XAFV/, and a detailed protocol was published in JMIR Research Protocols (53).

### 2.1 Eligibility criteria

Peer-reviewed empirical evaluation studies (qualitative, quantitative, and mixed-methods) published between 2014 and August 2024 were considered. Reviews, conference abstracts, study protocols, and development studies without prototype testing were excluded.

RPM interventions were eligible if they included a core symptom monitoring component that required active patient or caregiver engagement, for example, through manual data entry or confirmation, combined with integrated bidirectional interaction with healthcare providers. Fully automated systems that did not require active user engagement, such as passive monitoring via implants and sensors, were excluded.

We considered technologies intended for patients (adults, adolescents, and children) with cardiovascular disease or cancer. Studies with mixed or unspecified target populations were excluded.

Technologies implemented in or intended for clinical care settings were considered. Studies that used technology solely for collecting study data without any role in patient care were excluded. Studies conducted exclusively in inpatient settings were also excluded.

Studies published in English or German were eligible.

### 2.2 Search strategy

The search strategy combined terms related to “cancer or cardiovascular diseases,” “eHealth or telemonitoring,” and “evaluation designs” to identify relevant sources. In August 2024, we searched MEDLINE, Embase, CINAHL, PsycINFO, SocINDEX, and the Philosopher's Index for articles published from 2014 through August 2024. Google Scholar was used as a supplementary search source to identify additional peer-reviewed studies. The first 1,000 results were considered and deduplicated against records identified through the database searches before unique records proceeded to screening.

The full search strategies, including the database-specific search strings, are reported in the published scoping review protocol (53).

### 2.3 Study selection

All references were imported into EndNote 20 (Clarivate Analytics, PA, USA) and uploaded to Rayyan, an AI-powered web application for conducting systematic reviews (54). Rayyan automatically removed duplicates when similarity exceeded 95%. The first reviewer (LW) manually reviewed the remaining records. After removing duplicates, titles/abstracts and subsequently full texts were independently screened by the three reviewers (LW, JK, and SV), with each study receiving at least two decisions. Rayyan offers an AI-assisted tool that provides suggestions regarding whether records may meet the eligibility criteria. These suggestions did not determine final inclusion or exclusion decisions. Disagreements were resolved through discussion and consensus.

### 2.4 Data extraction

Study characteristic data (author, year, country, study design, participants, intervention type) were extracted using a structured form adapted from the JBI template (51).

The review-specific extraction tool was based on the draft tool described in the published protocol, which distinguished between ethically relevant issues related to the research process and those related to study outcomes. The tool and extraction criteria were refined during the review. The first 70 studies were independently extracted by two reviewers (LW and JK), who discussed discrepancies and refined the criteria. The remaining studies were extracted by LW, with uncertainties discussed with JK. The first 70 studies were then revisited and updated where necessary to ensure consistent application of the refined criteria. This differed from the published protocol, which had planned double extraction of all included studies.

Extraction focused on ethically relevant issues, which we defined as topics, practices, or findings with potential ethical significance, irrespective of whether the original authors explicitly framed them as ethical. Relevant passages were identified using four cues: (1) explicit references to ethics or ethical issues, supported by a keyword search for “ethic*”; (2) descriptions of negative, unintended, or ambivalent consequences of RPM; (3) normative or critically reflective statements, such as statements about what should, ought to, must, or needs to occur, or passages considering assumptions, perspectives, tensions, trade-offs, or consequences; and (4) practices or issues identified as ethically relevant in established digital health-specific ethical approaches [cf. (24, 29, 30, 34, 35)], including justice-related sampling practices and issues of responsibility, functionality, or usability. Identified passages were extracted verbatim together with their location in the manuscript.

### 2.5 Data synthesis

Study characteristics were summarized using descriptive statistics. Extracted ethically relevant passages were analyzed using basic qualitative content analysis in accordance with the JBI guidance (55).

A mixed deductive–inductive approach was applied. The process–outcome distinction prespecified in the protocol was further developed through open coding. Related codes were compared, merged, or differentiated and grouped into subcategories and broader categories. The developing category structure was discussed and iteratively refined by both reviewers (LW and JK). The resulting four analytical categories overlapped conceptually in some respects. To avoid multiple assignment of the same issue, each ethically relevant issue was assigned to the category that best reflected its primary ethical focus. For example, disadvantages arising from digital exclusion were categorized under Justice and Equity rather than also under Ambivalent Impacts of RPM. The final categories and corresponding subcategories are presented in Tables 2–5.

To characterize the extent of ethical engagement, defined here as how studies addressed ethically relevant issues, passages were additionally classified using the IS–OUGHT–ACTION structure. IS referred to descriptive reporting of an ethically relevant issue, practice, finding, or consequence. OUGHT referred to normative or critical reflection on an ethically relevant issue that moved beyond description. Explicit use of ethical theories, principles, or frameworks was not required for classification as OUGHT but was recorded when present. ACTION referred to proposed responses, changes, or recommendations related to the identified issue (see Supplementary Material 1 for illustrative examples of extracted material and its classification during the early phase of the analysis).

Analytical memos were recorded selectively to capture notable strengths or limitations in ethical engagement and recurring patterns across studies. During the analysis, candidate illustrative examples were identified and subsequently re-examined by both reviewers (LW and JK), who selected the final examples by consensus. Where appropriate, counts and percentages were calculated to provide an overview of how often a pattern occurred.

## 3 Results

### 3.1 Study selection and characteristics

Of the 4,648 initial records, 161 studies met the inclusion criteria after title/abstract screening and full-text reviews. The screening process and reasons for exclusion at the full-text stage are documented in the PRISMA flow diagram (Figure 2).

**Figure 2:**
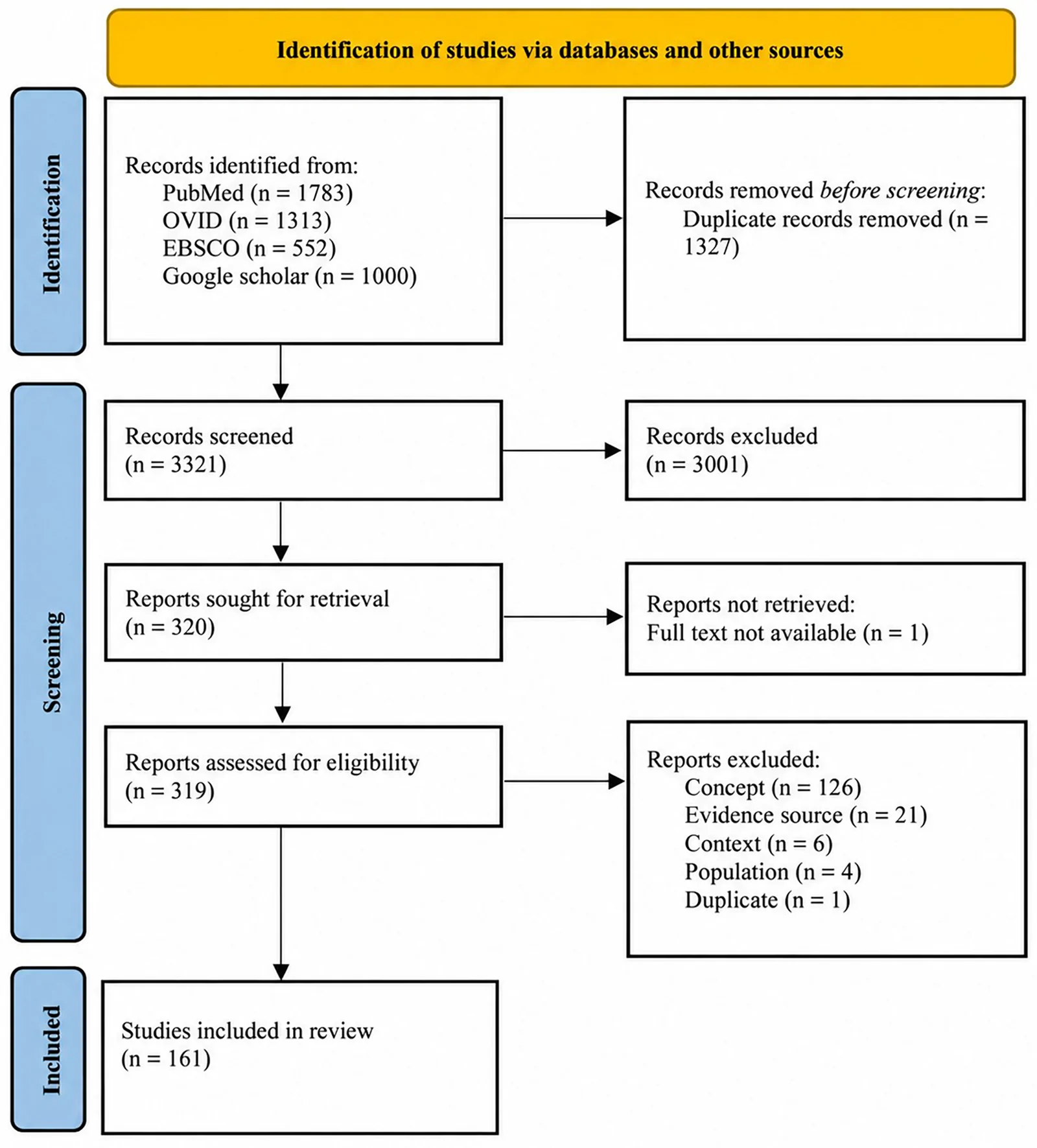
PRISMA flow diagram of the study selection process for this scoping review, adapted from the PRISMA 2020 flow diagram template (56).

Table 1 summarizes the key characteristics of the 161 included studies. The majority were published from 2020 onwards (*n* = 113; 70.2%), with most studies conducted in either North America (*n* = 61; 37.9%) or Europe (*n* = 60; 37.3%). Quantitative (*n* = 90; 55.9%) and mixed-methods designs (*n* = 52; 32.3%) dominated. Many were process-oriented and early-phase studies, including pilot and feasibility trials (*n* = 94; 58.4%), while 49 (30.4%) were full RCTs or sub-analysis of RCTs. Studies focusing on cancer (*n* = 90; 55.9%) were more frequent than those focusing on cardiovascular diseases (*n* = 71; 44.1%). Only five (3.1%) studies focused on children or adolescents. Most interventions combined symptom monitoring with additional features (*n* = 140; 87.0%), such as educational materials, chat functions, or pillboxes. Automated components, such as alerts or feedback based on entered health data, were common (*n* = 128; 79.5%), whereas the use of AI components was explicitly reported in only three (1.9%) studies. Further details of the study characteristics are provided in Supplementary Material 2.

**Table 1:** Study characteristics.

Category	Subcategory	Number (*n*)	Percentage (%)
Publication year	From 2020 onwards	113	70.2%
	Before 2020	48	29.8%
Geographical origin	North America	61	37.9%
	Europe	60	37.3%
	Asia	26	16.1%
	South America	7	4.3%
	Oceania	6	3.7%
	Multicontinental	1	0.6%
Methodology	Quantitative	90	55.9%
	Mixed-methods	52	32.3%
	Qualitative	19	11.8%
Study type	Process-/pilot studies (incl. development, usability, pilot RCTs, etc.)	94	58.4%
	RCTs (full or sub-analysis)	49	30.4%
	Other (e.g., case studies, cohort studies, economic evaluations)	18	11.2%
Target population	Cancer patients	90	55.9%
	Cardiovascular populations	71	44.1%
	Children/adolescents with cancer or cardiovascular disease	5	3.1%
Type of intervention	Symptom monitoring combined with other features	140	87.0%
	Symptom monitoring only	21	13.0%
Automated features	Alerts, reminders, feedback	128	79.5%
	Artificial intelligence components	3	1.9%

### 3.2 Findings in relation to the research questions

Explicit references to ethics were predominantly located in the Methods or Disclosure sections. These explicit references were limited to procedural aspects, such as ethics approval or informed consent procedures. Only one study referred to ethics in the Discussion section, noting that patients should be informed about RPM processes for “ethical reasons” (57). Another study employed a questionnaire in which ethics was included as one of six dimensions to assess expected and received knowledge of RPM (58). Furlong et al. (59) reported that, in addition to obtaining ethics approval, regular meetings were held to discuss ethical issues; however, no further details were provided. No explicit reference to “ethic” was identified in 29.8% (*n* = 48) of the included studies (60–107).

Ethically relevant issues without explicit ethical framing were identified in all studies, and across two or more of the four analytical categories developed: (1) *Integrity in Research and Development,* (2) *Ambivalent Impacts of RPM,* (3) *Justice and Equity,* and (4) *Data Privacy and Security* (further specified below). The first two categories were the most consistently represented across the sample. Overall, the depth of ethical engagement varied, ranging from brief, largely descriptive reporting of practices (e.g., measures to protect privacy) to normative or critical reflection on empirically derived findings (e.g., critical discussions of the ambivalent effects of RPM, such as simultaneously fostering patient empowerment and creating new forms of dependency).

The following sections describe the four categories and, within each, present findings on how authors reported their engagement with ethically relevant issues.

#### 3.2.1 Integrity in research and development

The category *Integrity in Research and Development* covers reported process-related aspects of research and software development. This included reporting on study approval, stated compliance with ethical guidelines and regulations, transparency measures such as disclosures, reported methodological rigor, measures to ensure participant well-being and minimize participant burden, as well as participatory research and software development practices (see Table 2 for details). All 161 studies reported at least one of these topics.

**Table 2:** Integrity-related practices in research and development.

Subcategories	Description
Study approval	Reporting on ethics committee or IRB approval status, including approval, exemption, or an explanation that formal ethics review was not required.
Stated compliance with ethical and regulatory standards	Statements of compliance with general research ethics guidelines (Declaration of Helsinki, Good Clinical Practice, National Ethical Guidelines for Epidemiological Studies in Japan), reporting of informed consent procedures, data protection laws and procedures.
Research transparency and disclosure practices	Reporting trial registration, funding disclosure, author contributions, conflicts of interest, commercial involvement, data availability, and related transparency measures.
Reported methodological rigor and reflexivity	Using validated tools or clearly reporting the use of non-validated tools, acknowledging potential sources of bias and outlining mitigation strategies, reflecting on design limitations and their impact on the interpretation of research findings, reporting missing or non-significant findings, and using reporting guidelines.
Safeguarding participant well-being and managing participation burden	Measures to safeguard participants’ psychological safety, reduce the burden of participation, report compensation for incurred expenses, or exclude vulnerable patients due to safety concerns.
Stakeholder involvement in research and tool development	Emphasizing or conducting stakeholder involvement in conceptualizing the research or developing the eHealth tool, for instance, involving patient representatives in shaping the research focus or clinicians in developing algorithms.

A notable finding across the reviewed studies concerned variations in reported approval processes: 92.5% (*n* = 149) of the reviewed studies reported on a study approval process, with some referring to approval by an ethics committee (50, 57–59, 95, 108–165), and some to approval by an institutional review board (IRB) (61, 63, 64, 68, 71, 73, 76, 78, 81, 83, 84, 88, 90–93, 95, 97, 99–102, 104, 106, 108, 165–180). In two studies, reference was made to a research committee, leaving the nature of the committee unclear (60, 181). In some cases, alternative authorities were involved in the approval process, such as the Spanish Agency of Medicines and Medical Devices (110) or data protection authorities (182, 183). A minority of studies stated that formal ethics committee or IRB approval was not required, or that a waiver of informed consent had been granted. These decisions were justified by classifying the study as minimal risk, framing it as a quality improvement initiative, or citing relevant national legislation (134, 172, 177, 184, 185). In 7.5% (*n* = 12) of studies, no information on ethical or IRB approval could be identified (62, 65, 66, 77, 80, 82, 86, 89, 94, 98, 107, 186); this does not necessarily mean that study approval was absent, but rather that it was not reported explicitly.

In addition to variations in reported approval processes, the publications provided limited information on which ethical issues were considered during ethics review. Most reports provided only minimal, checklist-style information, usually stating that ethical approval and informed consent had been obtained. However, we also identified studies that provided more detailed information, for instance, on informed consent procedures, participants' rights to withdraw, measures to ensure the confidentiality of participant information, restricted access to study data, and reflections on design-specific challenges, such as power imbalances in interview settings (136, 140, 150, 154, 187, 188). Ethical issues specific to digital health contexts were rarely discussed explicitly in the Ethics or Ethical Considerations sections. The topics addressed included reporting on clinician compensation for RPM-related work and assurances of flexible scheduling (57, 150, 163, 189). Challenges related to informed consent in digital health were also addressed in one study, which noted difficulties in understanding system interfaces and associated risks; in response, visual materials illustrating the RPM system were incorporated into the informed consent process (174). Another study provided a detailed description of data protection measures for both the study data and digital health tool (134).

Another notable pattern was that we did not identify references to digital health-specific ethical frameworks guiding the conduct of evaluation studies. In contrast, general health research ethics guidelines were frequently cited, most commonly the Declaration of Helsinki and Good Clinical Practice guidance (78, 110, 112, 114, 117, 125, 128, 130, 133, 137, 140, 143, 145, 146, 149, 150, 152, 156, 158, 161, 165, 168, 175–178, 180, 190–192). Other references included the National Ethical Guidelines for Epidemiological Studies in Japan (157), and the ethical guidelines for biomedical research involving human subjects at Ningxia Medical University (193), neither of which is specific to digital health. Guidance more directly related to digital health focused primarily on reporting, implementation, or legal requirements rather than on ethical frameworks.

These included reporting standards, such as the Consolidated Standards of Reporting Trials (CONSORT)—EHEALTH guideline (100, 110, 154, 167, 180, 194, 195), the Holistic Framework for the Uptake and Impact of e-Health Technologies (196), and compliance with data protection regulations (140, 151, 152, 158, 180, 182, 183, 191).

Reflective practice was rarely identifiable in sections reporting ethics approval or ethical considerations. This pattern also extended to the disclosure sections, in which industry involvement through study funding, honoraria, or author employment was reported (50, 59, 63, 66, 75, 83, 85, 87, 91, 95, 103, 106, 110, 111, 114–116, 123, 125, 132, 134, 138, 140, 141, 147, 151–153, 155, 156, 158, 170, 172, 179, 185, 191, 195, 197–200). In 61% of these studies (*n* = 25 of 41), industry involvement was merely disclosed, without further information on the role or involvement of commercial actors in study design, conduct, or reporting (59, 63, 75, 83, 85, 87, 103, 106, 111, 115, 116, 125, 132, 141, 147, 151–153, 156, 158, 172, 191, 195, 198, 200). The remaining studies provided additional information about the role or involvement of commercial actors, although often only briefly; we did not identify substantive critical reflection on the implications of this involvement. In one case, the involvement of a conflicts of interest committee was reported, although no further details were provided regarding its function (100).

#### 3.2.2 Ambivalent impacts of RPM

The category *Ambivalent Impacts of RPM* includes outcome-related ethically relevant issues concerning the (potential) effects of RPM. This encompasses the positive and/or negative impacts that may affect individuals, their interpersonal relationships, or broader societal contexts. In contrast to the previous category, which focused on research process-related issues, this category centered on the outcome-related dimensions of RPM.

The category comprises the following subcategories: effects on patient health outcomes, including clinical benefits and/or burdensome effects; impacts on patient and caregiver autonomy, ranging from greater self-management to increased dependence on clinician feedback; communication, relationships, and care orientation, reflecting tensions between connection and detachment; professional roles and decision-making, indicating both support and open questions regarding accountability; resources, workload, and economic implications, encompassing efficiency as well as strain; and technical performance, data quality, and trust, ranging from reliable RPM performance to safety issues due to technical errors and alert fatigue (see Table 3).

**Table 3:** Positive and/or negative impacts of RPM.

Subcategories	Description
Patient health outcomes and perceptions (benefits vs. Burdens)	Potentially beneficial and/or burdensome effects of RPM, including improved health outcomes and perceived safety, as well as anxiety, overwhelm, increased care burden, limited perceived value, and altered illness perceptions.
Patient and caregiver autonomy (empowerment vs. Dependency)	Empowering patients through transparent data access and shared decision-making, contrasted with the risks of paternalism, over-reliance on the care team, or a lack of motivation to engage in self-care.
Communication, relationships, and care orientation (connection vs. Detachment)	Effects of RPM on clinician-patient relationships, including enhanced communication and patient-centered care, and/or risks of more alert-driven, less personal, or disease-centered interactions and concerns about burdening clinicians.
Professional roles, decision-making, and accountability (support vs. Constraint)	Tensions between algorithm-based decision support and professional judgement, alongside shifts in roles and responsibilities that may enhance coordination and efficiency but also create over-reliance, role conflicts, accountability gaps, and uncertainty about responsibility for RPM data.
Resources, workload & costs (efficiency vs. Strain)	Resource implications of RPM, including improved efficiency, access to care, and reduced healthcare costs, alongside increased workload, new tasks, staffing challenges, limited reimbursement, and financial burdens for professionals and patients.
Technical performance & trust (reliability vs. Risks)	Technical and data-related aspects of RPM that may facilitate or hinder use, including usability and functionality, integration and connectivity challenges, and the benefits and risks of alert systems (e.g., timely insights and reassurance versus false alerts, alert fatigue, data integrity concerns, and reduced trust).

All reviewed studies reported at least one of the topics represented in the subcategories at a descriptive level, but three central gaps in ethical engagement were identified. First, we did not identify an explicit reference to an ethical approach used to guide reflections on observed RPM impacts. Second, when problems, challenges, errors, and tensions with ethical relevance were reported in full texts, these issues were not explicitly framed or discussed as ethical concerns. Instead, they were solely acknowledged at the empirical level, for example, as issues of functionality, implementation, adherence, acceptance, or efficacy. Third, a consistent imbalance in the reporting of RPM impacts was evident. While we identified reporting of positive impacts in all studies and across different manuscript sections, negative aspects were addressed less systematically and in less depth. The following examples illustrate this pattern: In 31.7% (*n* = 51) of the reviewed studies, little to no critical perspective on the impacts of RPM was evident in the full text, with critical reflections largely confined to methodological limitations (65, 68, 69, 74–76, 83–85, 89, 91, 94, 97, 103–105, 107–110, 114, 115, 123, 127, 129, 131, 133, 135, 138, 140, 142, 143, 146, 152, 155, 156, 158, 159, 161, 162, 176, 181, 186, 187, 191, 194, 199, 201–204). In 79.5% (*n* = 128) of the studies, we did not identify negative aspects in the abstracts, or these were mentioned only very briefly or framed positively (57, 59–76, 78, 79, 82–87, 89–91, 93–99, 103–111, 113–124, 127, 129–136, 138–143, 145–156, 158–166, 170–173, 176, 178–181, 183–187, 189, 191, 193–197, 199, 201–212). This included both studies with little to no critical perspective in the full text and studies in which critical perspectives on RPM were identified.

We also identified illustrative examples that, despite lacking explicit ethical framing, presented a more balanced representation of positive and negative impacts. These examples were mainly found in process-oriented study designs, and commonly used qualitative or mixed-methods approaches. Two of these studies foregrounded critical perspectives: a lessons-learned study by Russell et al. (112) and an implementation feasibility study by Seto et al. (213). Within the process-oriented sample, we identified studies in which participants were encouraged to critically reflect on their experiences (61, 70, 118, 119, 122, 168, 169, 174, 198, 214).

In several studies using process-oriented designs, positive and negative aspects were often presented in direct contrast. For example, RPM was described as providing patients with psychological reassurance while also contributing to care burden, changes in illness perceptions, stress, and anxiety related to continuous monitoring, and concerns about the clinical value of certain alerts (58, 59, 81, 118, 157, 160, 174, 188–190, 196, 213). Another example related to patient and caregiver autonomy, as findings highlighted tensions between enhanced self-management and increased dependence on clinical feedback, as well as patients' inability to self-manage their conditions and discomfort associated with perceived surveillance and impersonal technologies (57, 137, 160, 177, 188, 190, 192, 213). In terms of communication, relationships, and care orientation, studies highlighted tensions between efficiency and relational continuity. Findings pointed to shifts from person- to problem-centered care and increasing reliance on automated processes, alongside the need to maintain human involvement and shared decision-making (147, 153, 188, 214, 215). Issues related to professional roles—often intended to be strengthened and supported through RPM—included role ambiguity, limited decision-making authority, and tensions arising from the co-management of patients across professional groups (120, 122, 130, 144, 157, 212, 213). Although resources, workload, and economic implications were frequently presented as key benefits of RPM, several studies also highlighted its resource-intensive nature and associated sustainability concerns. These include challenges related to staffing, increased costs, and greater workload, for example, due to the responsibility for alert management, documentation demands, and substantial technical support requirements (58, 59, 81, 118, 173, 185, 196, 213, 216). Finally, while many studies reported favorable technical performance, others reported system integration challenges, data integrity concerns, non-actionable alerts resulting in alert fatigue and reduced trust, usability problems, confusing educational content, and navigation difficulties (57, 59, 61, 118, 119, 122, 160, 168, 169, 174, 196, 198, 213, 214).

While most insights into tensions and ambivalence originated from process-oriented studies with qualitative design components, some RCTs also addressed these ethically relevant issues (63, 71, 84, 90, 100, 102, 106, 113, 167, 170, 175). However, these were examined in a less thorough and systematic manner, typically confined to the Discussion sections, and often based on findings from other studies. As in process-oriented studies, we did not identify explicit ethical framing of these issues.

#### 3.2.3 Justice and equity

We clustered a mix of process- and outcome-related issues concerning *Justice and Equity* into the following subcategories: equity-oriented research methodologies, systemic and individual barriers to equity, and mitigation strategies and inclusive design (Table 4).

**Table 4:** Justice and equity-related practices.

Subcategories	Description
Equity-oriented research methodologies	Methodological practices and study designs aimed at transparently capturing and addressing inequalities. This involves assessing digital literacy and technology access as baseline variables, reporting transparent exclusion metrics (e.g., documenting non-participation or dropouts due to technological or emotional hurdles), conducting subgroup analyses across demographic characteristics, employing equity-driven qualitative sampling strategies (e.g., purposive or maximum variation sampling to ensure representation of diverse, vulnerable, or historically marginalized groups).
Systemic and individual barriers to equity	Reporting or discussing barriers that may hinder access to and use of RPM. This encompasses infrastructural and geographic barriers (e.g., lack of broadband internet, financial constraints, or residing in rural areas) as well as patient-specific vulnerabilities (e.g., advanced age, cognitive or physical limitations, language barriers, and low digital or health literacy).
Mitigation strategies and inclusive design	Active clinical, technical, and operational measures taken to overcome identified access barriers and promote inclusivity. This includes the provision of hardware or software (e.g., facility-provided tablets), the implementation of low-tech alternatives (e.g., automated interactive voice response telephone calls or SMS), the delivery of targeted technical training and human support, and inclusive user-centered design and usability testing aimed at improving accessibility prior to implementation.

More substantive engagement with issues of equity and justice in digital health was identified in only a minority of studies (30.4%; *n* = 49). These studies incorporated equity and justice considerations into their design and/or acknowledged in more than a brief or incidental manner that digital health interventions may reinforce existing health disparities. In some cases, these concerns were explicitly framed in terms of concepts such as inequality, disparities, and the digital divide (50, 58, 63, 75, 81, 83, 87, 88, 90–93, 98–100, 102, 106, 108, 113, 117, 120, 124, 125, 130, 132, 136, 137, 148, 149, 157, 163, 165, 166, 168, 171, 174, 175, 179, 182, 184, 188, 192, 198, 200, 207, 210, 214–216).

Practical measures aimed at reducing access barriers and promoting inclusivity were also frequently reported. Reported approaches included the provision of mobile devices, reimbursement of internet costs, mobile hotspots, multilingual content, technical support, and paper-based alternatives (50, 61, 63, 77, 79, 87, 89, 93, 99, 100, 102, 104, 106, 109, 117, 118, 121, 126, 133, 139, 157, 159, 163, 166, 167, 170, 175, 176, 178, 195, 197, 212–216). In some studies, multiple approaches were combined. For example, Dang et al. (99) targeted minority populations through language adaptation, device provision, and technical support, while Shara et al. (93) tailored their intervention to underserved groups with low socioeconomic status and limited literacy while also reflecting on remaining exclusions. Other studies implemented or recommended inclusive design features that address the needs of older adults (50, 171, 210) or ethnic minority populations (168). Within the research process, justice considerations were operationalized through inclusive recruitment and study design. Basch et al. (106) increased recruitment of Black/African American participants, conducted subgroup analyses, and provided multilingual access in English, Spanish, and Mandarin.

The relatively small number of studies demonstrating substantive engagement with equity and justice issues contrasts with the large number of reported ineligible patient groups, participant withdrawals, and study dropouts. For example, Girgis et al. (130) reported that nearly half of their target population was ineligible. Across the reviewed studies, groups identified as being at particular risk of exclusion or non-benefit included older adults, individuals with limited digital competence, and people with physical impairments (50, 58, 61, 76, 77, 80, 91, 93, 94, 98, 101, 109, 116, 117, 120, 122, 125, 126, 130, 151, 153, 155, 159, 169, 171, 174, 175, 177, 190, 192, 206, 210, 214, 215). Further groups included socioeconomically disadvantaged populations (63, 78, 81, 83, 87, 93, 95, 98, 99, 113, 117, 120, 128, 130, 136, 151, 157, 163, 168, 175, 180, 200, 212, 214), and rural communities facing geographic inequities (70, 88, 94, 98, 105, 107, 121, 151, 162, 167, 174, 182, 187).

When focusing specifically on the reporting of exclusions and patterns of potential non-benefit, we found limited explicit consideration of equity and justice in the majority of the reviewed studies (68.3%; *n* = 110). Such considerations were either not identified, mentioned only briefly, or framed primarily as methodological limitations related to selection bias and limited generalizability (57, 59–62, 64–74, 76–80, 82, 84–86, 89, 94–97, 100, 101, 103–105, 107, 109–112, 114–116, 118, 119, 121–123, 126, 127, 129, 131–133, 135, 138–147, 150–156, 158–162, 164, 167, 169, 170, 172, 173, 176–178, 180, 181, 183, 185–187, 189–191, 193–197, 199, 201, 203–206, 209, 211, 213, 214).

#### 3.2.4 Data privacy and security

The category *Data Privacy and Security* included a mix of process- and outcome-related ethically relevant issues. These encompassed procedural descriptions of data management and safeguards concerning the digital health tool, statements of compliance with data protection regulations, approval processes and contractual arrangements, reported data privacy concerns expressed by patients, caregivers, and clinicians, as well as explicit discussions of privacy and security challenges (see Table 5 for details).

**Table 5:** References to data privacy and security-related practices.

Subcategories	Description
Procedural descriptions of data management	Descriptions of technical safeguards such as data access control, secure data storage, encryption, authentication, or penetration tests to protect patient data. Information on who manages the data, how it is stored, who has access, often reported in Methods section.
Compliance with data protection regulations	Statements of compliance with data protection regulations and formal approvals from data protection authorities, and data processing contracts.
Data privacy concerns	Reporting users’ concerns about data privacy and security in home monitoring, including concerns about being “spied on”; this also included calls for researchers to be sensitive to these issues and reports identifying privacy as a central patient need.
Discussing privacy & security challenges	Studies that go beyond description to critically examine the difficulties of implementing robust data protection in eHealth and highlight regulatory and technical challenges.

Reflective engagement with *Data Privacy and Security* was uncommon (6.2%; *n* = 10). The few studies identified explored privacy perceptions among patients and caregivers in home monitoring settings (174, 180), accounts of RPM as experienced as both supportive and intrusive (192, 211), and the identification of privacy protection as a central patient need in digital health (150). One study highlighted a data protection shortcoming, noting that images stored on patients' personal devices remained unprotected outside the app environment (113). Others described tensions between stringent data security requirements and resulting limitations in system integration, clinical utility, and usability (87, 119, 128). Zheng et al. (179) identified data protection as a key implementation challenge, linked it to relevant regulatory frameworks, and underscored the importance of privacy and trust in digital care. None of these studies explicitly framed these issues as ethical concerns.

Studies providing comprehensive descriptive accounts of data privacy and security were also rare (14.9%; *n* = 24). For instance, illustrative studies provided detailed descriptions of measures, such as encryption, access controls, authentication procedures, data storage, and anonymization (67, 70, 77, 124, 128, 131, 134, 154, 157, 181, 184). Other studies reported data processing agreements and broader data governance or regulatory oversight mechanisms, including independent data monitoring committees and review by regulatory authorities, such as the U.S. Food and Drug Administration and the Spanish Agency of Medicines and Medical Devices (79, 87, 88, 91, 92, 110, 113, 128, 131, 138, 143, 152, 179, 184, 215). Indraratna et al. (108) reported external penetration testing and formal registration of their application as a medical device. The involvement of data protection or regulatory authorities provides an indirect indication that data privacy and/or security requirements may have been considered at some stage of the research process or technology lifecycle, although the specific issues addressed were not explicitly reported in the manuscript.

In contrast to these illustrative examples, most studies (82.6%; *n* = 133) provided little or no information on data privacy and security measures related to the digital health tool evaluated. Where reported, descriptions were often superficial or vague (50, 57, 58, 60–66, 68, 69, 71–76, 78, 80–86, 89, 90, 93–107, 109, 111, 112, 114–118, 120–123, 125–127, 129, 130, 133, 135–137, 139–142, 144–151, 153, 155, 156, 158, 159, 161–178, 180, 182, 183, 185–214, 216). For instance, several studies referred to systems as “secure” or “highly confidential” without providing further details or explanation (58, 75, 81, 87, 90, 94, 97, 101, 132, 136, 139, 141, 143, 144, 146, 148, 149, 156, 158, 167, 176, 180, 194, 196, 205, 212, 213). In some cases, authors referred readers to external sources, such as websites, previously published protocols, development studies, or trials (57, 61, 101, 103, 105, 130, 136, 139, 141, 145, 148, 160, 164, 165, 174, 183, 195, 196, 200, 202, 207, 214). While the review of these external documents was not a focus of this scoping review, these documents may provide information on security features and measures.

## 4 Discussion

This scoping review examined how ethical issues are addressed and reported in original digital health evaluation studies, using RPM in cancer and cardiovascular care as illustrative cases. By analyzing text passages that explicitly state or imply ethical relevance, this review provides insights into the extent, depth, and methodological handling of ethical issues.

### 4.1 Principal findings

We developed four analytical categories that, while not identical, broadly align with the ethical domains described in existing digital health ethics frameworks [cf. (3, 24, 29, 30)] and collectively encompass all four principles of biomedical ethics (23). Across these categories, we found limited reporting of systematic, explicit, and digital health-specific ethical reflection, consistent with findings from previous reviews (42, 45, 47, 48, 217). However, we also identified examples of more substantive ethical engagement, in which concrete measures or reflective practices concerning ethically relevant issues were evident in the reporting, which we present as illustrative examples.

#### 4.1.1 Procedural reporting without substantive, digital health-specific ethical reflection

In *Integrity in Research and Development*, brief statements indicating that procedural requirements had been fulfilled predominated over reporting ethical reflection. For example, approval by an ethics committee or IRB was reported in 92.5% of studies. However, little information was provided on which ethical issues were considered during these reviews. References to digital health-specific ethical frameworks were not identified, a gap also identified by Gooding and Kariotis et al. (42). A similar pattern was evident in disclosure sections, particularly for conflicts of interest, which rarely went beyond descriptive reporting.

These findings reveal a gap between reporting that procedural requirements were met and making the underlying ethical reflection transparent. Without further information, readers cannot assess which ethical issues were considered or how thoroughly they were examined (42). A similar concern applies to conflicts of interest. Simply disclosing relationships with technology or pharmaceutical companies does not allow readers to assess their potential influence on the research or how potential conflicts were addressed (218). This is particularly relevant given the growing role of commercial actors in digital health. Limited transparency may obscure commercial influences on research priorities, study conduct, and the interpretation of findings (30, 219, 220).

Beyond the observed lack of transparency, another finding was that we did not identify reports of systematic ethical approaches extending beyond the context of ethics approval. Ethics approval focuses primarily on the study conceptualization phase, while issues emerging throughout the research process are typically not covered (221). It therefore remains unclear whether and how such emerging ethical issues were addressed. Reliance on ethics committees and IRBs may also have limitations because their expertise and procedures vary (42, 222–224). For instance, if only general research ethics standards such as the Declaration of Helsinki are considered in these procedures, ethical issues specific to digital health research may not be adequately captured (11, 223, 225, 226).

#### 4.1.2 Imbalanced reporting and limited ethical framing of outcomes

Within *Ambivalent Impacts of RPM*, the central gaps were an imbalance between positive and negative perspectives and limited systematic reflection on the ethical implications of outcomes. Despite the broad range of ethically relevant issues identified across patients, caregivers, professionals, and health systems, negative perspectives received less attention than positive ones. This imbalance was particularly evident in the abstracts: in 79.5% of studies, negative aspects had limited visibility. Moreover, when negative impacts were reported, they were typically discussed as empirical findings rather than explicitly examined as ethical issues.

Our findings align with broader evidence of limited attention to negative effects in digital health research (227, 228). Digital health-specific reporting guidance explicitly calls for the reporting of harmful effects; however, this was not consistently reflected in the reviewed studies. This imbalance in reporting is not specific to digital health research. For example, Junqueira et al. (229) showed that, despite the widespread use of the CONSORT reporting guideline, its extension for reporting harms remains underused.

More balanced approaches were evident in process-oriented studies, particularly those using qualitative designs. These studies addressed a broader range of ethically relevant issues, consistent with literature suggesting that qualitative approaches are well suited to ethical analysis (33, 36, 221, 230). However, some quantitative studies also included ethically relevant issues in their discussions. Ethical reflection on outcomes therefore appears feasible across study designs, while we acknowledge that word limits can constrain the level of detail. It may be particularly valuable in outcome-focused trials, such as RCTs, by placing their findings within a broader societal context (21).

Limited attention to negative impacts can have consequences beyond individual studies. Inconsistent consideration and reporting may contribute to an overly optimistic evidence base and misguide policy and implementation decisions (231). Balanced reporting is therefore important for assessing the benefits, risks, and potential trade-offs of digital health. Insufficient attention to negative impacts may also hinder harm prevention and opportunities to learn from errors, thereby slowing innovation (15).

#### 4.1.3 Procedural reporting with limited ethical reflection on exclusion

Within *Justice and Equity*, the central gap was limited ethical reflection on study exclusions and their implications for equitable healthcare. Although extensive exclusion criteria were reported, 68.3% of studies did not discuss their implications beyond methodological limitations. Some studies, however, explicitly framed these concerns in ethical terms, making issues of justice and equity more visible. Together with reported efforts to reduce digital barriers, these findings are consistent with the increasing recognition of equity-related challenges in digital health (232), although substantial gaps remain in how these challenges are addressed in evaluation studies.

This limited ethical reflection has important implications for health equity, as digital health interventions intended to reduce disparities may inadvertently reinforce them if appropriate countermeasures are not implemented (21, 44, 45, 233, 234). Our findings are consistent with Poli et al. (235), who identified selective participation as an equity concern in digital health research. Older, frailer, and less digitally skilled individuals were less likely to participate. Their underrepresentation may bias study findings and produce evidence that does not adequately reflect the needs and outcomes of vulnerable groups.

However, exclusion is not necessarily inappropriate. It may be justified by feasibility or safety considerations and, in some cases, may be ethically warranted. This creates a potential ethical risk–benefit dilemma when populations excluded for their protection are also among those who may benefit most from digital health interventions. The ethical issue is therefore not exclusion *per se*, but how the reasons for exclusion are balanced against its potential consequences for equity (232, 236). Making this trade-off explicit would allow readers to assess not only the methodological justification for exclusion but also its implications for equity.

#### 4.1.4 Vague security claims and limited ethical reflection

In 82.6% of studies, little or no information on digital health specific *Data Privacy and Security* measures was identified. In some cases, authors described tools as “secure” without providing technical, procedural, or organizational details to support these claims. However, some studies provided detailed descriptions of technical safeguards, demonstrating that more comprehensive reporting is feasible. Even in these studies, reflection on the ethical implications of data privacy and security was rare.

This limited reporting is notable because data privacy and security are widely recognized ethical concerns in digital health (45, 237). Moreover, established reporting standards, such as CONSORT-EHEALTH and mERA, already require related information (238, 239). However, these digital health-specific guidelines were seldom referenced, which may partly explain the limited reporting observed. Vague claims of security also raise questions about whether researchers across disciplines and national contexts share a common understanding of what constitutes adequate security (240). As with ethics approval and conflicts of interest, such statements are difficult to evaluate. Without sufficient detail, readers cannot assess how privacy and security were addressed or determine whether claims that a technology is secure are adequately supported.

#### 4.1.5 Cross-category pattern and potential explanations

Ethics approval and conflicts of interest were documented without substantial reflection; negative impacts were reported less consistently and rarely examined through an ethical lens; exclusions were primarily discussed as methodological limitations; and privacy and security were often omitted or addressed only at a descriptive or technical level. Across these categories, ethically relevant issues were not absent, but their ethical implications often remained unexplored.

Limited visibility of ethical engagement in empirical research is not a new concern (41), and several factors discussed in the literature may contribute to this pattern. One potential factor relates to established conventions and structures of empirical research. Reporting standards, methodological guidelines, and journals generally do not require in-depth ethical reflection or transparent reporting of such reflection (241). Ethically relevant issues may also be addressed primarily within established methodological categories, while explicit ethical analysis may fall outside researchers' usual disciplinary approaches (41). Word limits and expectations to remain closely focused on the study topic may further restrict the reporting of broader ethical discussion (242). Depending on the research paradigm, normative ethical reflection may also be perceived as difficult to reconcile with the aim of maintaining objectivity, particularly within positivist approaches (221).

A second factor is the complexity and resource requirements of ethical analysis. Identifying ethically relevant issues can itself be challenging (243). Moreover, there is no single agreed-upon ethical approach that is suitable for all digital health settings. Different frameworks and perspectives may need to be selected, adapted, or combined depending on the context. This process requires ethical expertise, time, and resources that may not always be available within existing research budgets and funding structures (26).

Finally, academic incentives may influence the extent and balance of ethical engagement. Scientific success is closely linked to publications and citations, while positive findings are more likely to be published and cited (231, 244). These incentives may contribute to greater emphasis on positive findings than on risks, limitations, and negative consequences, potentially discouraging balanced ethical reflection.

### 4.2 Recommendations to strengthen ethical engagement

As Hofmann et al. (41) argued in the context of Health Technology Assessment, such barriers do not justify the omission of ethical considerations. Based on our findings, we therefore propose concrete recommendations to strengthen ethical engagement in original digital health evaluation research.

- Consider digital health-specific ethical issues throughout the research process: Brief statements confirming formal ethics or IRB approval should be complemented by transparent reporting of the key ethical issues addressed during both the ethics/IRB review and the subsequent research process. Beyond general research ethics standards, ethical issues specific to digital health should be explicitly considered. Relevant information can be incorporated into Ethics or Methods sections or provided through references to openly accessible documents, such as study protocols or supplementary materials.
- Balance the consideration of digital health impacts and reflect on their ethical relevance: Evaluations should give balanced attention to positive and negative impacts of digital health interventions. Digital health-specific ethical frameworks may help researchers identify ethically relevant issues emerging from empirical findings and support more systematic ethical reflection. Such reflections can, for instance, be incorporated into Discussion sections. Explicitly framing these aspects as ethical can increase the visibility of ethical engagement, facilitate reviews and synthesis of ethical issues, and strengthen the contribution of empirical research to broader ethical debates in digital health.
- Extend descriptive reporting of justice and equity through ethical reflection: Given the risk of inequities associated with digital health interventions, exclusions and dropouts should be critically examined for their ethical implications and, where applicable, accompanied by applied or proposed mitigation strategies. Relevant reflections could be incorporated into Methods, Discussion, Limitations, or disclosure sections. This may strengthen awareness of inequity risks and support the development of more effective mitigation strategies.
- Explicitly address data privacy and security, including unresolved challenges: Research teams should transparently report how data privacy and security issues are addressed or clearly reference openly accessible sources containing this information. Remaining challenges and their ethical implications should also be critically discussed. Such transparency supports shared learning across development, research, and implementation contexts and may contribute to safer and more trustworthy digital health practices.

Although these recommendations may initially appear to place responsibility primarily on researchers, their implementation requires contributions from different actors and supportive structures.

Clinicians, caregivers, patients or public representatives can help identify burdens, exclusion, and unintended consequences, informing study design, interpretation, and reporting. Software developers can provide detailed information on privacy, security, functionality, and unresolved technical limitations. They can also contribute to reflecting on the implications of related design decisions. Ethicists can bring together perspectives from different actors and facilitate joint critical reflection throughout the research process. This can help ensure that digital health-specific ethical issues are addressed and that the resulting reflections are made transparent in study reports (32, 33).

Beyond individual research projects, educational institutions can strengthen researchers' ability to identify and critically examine ethically relevant issues specific to digital health (3, 28, 221), while funding bodies can provide necessary resources for ethical expertise and interdisciplinary collaboration (245). Ethics committees can encourage consideration of digital health-specific ethical issues during protocol review and help identify issues that warrant continued ethical reflection and transparent reporting in manuscripts (3, 223). Where possible, the ongoing involvement of ethicists within research teams can extend this support beyond the initial review and throughout the research process. Journals and peer reviewers can also promote transparent ethical engagement, for instance by encouraging balanced reporting of positive and negative findings, and corresponding critical reflection on ethically relevant issues. This also includes reflective practice concerning issues related to conflicts of interest (229, 241). The development of shared empirical-ethical guidance could further support this process by clarifying when descriptive reporting is sufficient, when further ethical reflection is warranted, and how this reflection and any resulting responses should be reported.

More transparent ethical engagement could allow insights from real-world evaluations to inform broader ethical debate. This is especially relevant in digital health, where ethical guidance may struggle to keep pace with rapidly evolving technologies and has been criticized for being disconnected from practice (15, 33, 245).

### 4.3 Strengths and limitations

A key strength of this review is the interdisciplinary composition of the review team, which included empirical researchers, clinicians, software developers, and ethicists, reflecting the complexity and cross-disciplinary nature of the review questions.

The findings and recommendations of this review do not constitute a comprehensive ethical framework or a complete list of ethical issues related to digital health evaluation. Instead, they aim to inform methodological discussions and support the development of practical, shared empirical-ethical guidance.

As ethically relevant text passages did not require an explicit reference to ethics, their identification required a certain degree of interpretation. Identification was further complicated by the fact that engagement with ethically relevant issues was sometimes distributed across multiple manuscript sections. There is therefore a risk that brief or isolated statements and reflections may have been overlooked, whereas sustained and explicit engagement was less susceptible to this limitation. Numbers and frequencies reported in this review should therefore be interpreted in light of these limitations.

Moreover, our analysis was limited to what was reported in the included publications. The absence of explicit ethical reflection in a study report does not necessarily indicate that such reflection did not occur during the research process. Additional limitations concern the generalizability and temporal scope of the results. The included studies focused on non-fully automated RPM used outside inpatient settings in cancer and cardiovascular care, which may limit transferability to other contexts. Furthermore, despite a comprehensive search strategy, some relevant studies may have been missed.

The search was completed in August 2024 and therefore does not capture subsequent developments, particularly in AI-enabled RPM, which was represented by only a few studies in our sample. However, in contrast to the rapidly evolving technological landscape, the integration of ethical reflection into evaluation practice appears to remain a persistent challenge across the literature (41, 48, 217). The search date is therefore more likely to affect the coverage of specific ethical implications associated with newer technologies than the broader pattern of ethical engagement identified in this review.

## 5 Conclusion

Ethically relevant issues were evident throughout digital health evaluations of non-fully automated RPM in cancer and cardiovascular care. However, they were often addressed as procedural or empirical matters rather than critically examined for their ethical implications. Across the four categories, we found limited reporting of explicit and systematic ethical reflection, alongside a tendency to give less attention to negative aspects.

A key question when reporting and reviewing digital health evaluations is therefore not simply whether ethically relevant issues concerning digital health are described, but whether their ethical implications are critically examined and made visible in the reporting. Our recommendations aim to strengthen the integration of ethical reflection into digital health evaluation. Alongside interdisciplinary collaboration and supportive institutional structures, we advocate for the development of shared empirical-ethical guidance across study designs to strengthen the visibility of ethical engagement in future digital health research.

Bridging the gap between empirical evaluation and ethical reflection may support more ethically robust digital health research and contribute to more ethically informed technologies and their use in practice.
